# Retraction Note: Prediction of intraperitoneal adhesions using striae gravidarum and scar characteristics in women undergoing repeated cesarean sections

**DOI:** 10.1186/s12884-025-08159-x

**Published:** 2025-09-15

**Authors:** Mohamed Elprince, Omima T. Taha, Zakia M. Ibrahim, Rasha E. Khamees, Mahmoud A. Greash, Khaled A. Atwa, Ahmed M. Gadallah, Noha al-Okda, Radwa M. Abdel Aal, Mohamed F. Ibrahim, Ahmed A. Aboelroose, Osama E. Ashour, Asmaa M. Elgedawy, Amira M. Elbahie, Hanan M. Ghoneim, Amal A. Ahmed, Amal A. Ahmed

**Affiliations:** Department of Obstetrics and Gynecology, Faculty of Medicine, Suez Canal University, Ismailia, Egypt

**Retraction note: BMC Pregnancy and Childbirth 21, 286 (2021)**

**https://doi.org/10.1186/s12884-021-03763-z**

The Editor has retracted this article. After publication of this article, concerns were raised about the plausibility of the data. The authors failed to provide their original data on request. The Editor has therefore lost confidence in the integrity of the results reported in this article. The authors did not state explicitly whether they agree to this retraction.

